# Cyanidotris(1,3,5-tri­aza-7-phosphaadamantane)silver(I) tetra­hydrate

**DOI:** 10.1107/S2414314625009253

**Published:** 2025-10-24

**Authors:** Sizwe J. Zamisa, Adesola A. Adeleke, Orpah Zinyemba, Bernard Omondi

**Affiliations:** a School of Agriculture and Science, Discipline of Chemistry, University of KwaZulu-Natal, Private Bag X54001, Durban, 4000, South Africa; b Department of Chemical Sciences, University of Johannesburg, PO Box 524 Auckland, Park, Johannesburg, 2006, South Africa; University of Antofagasta, Chile

**Keywords:** crystal structure, 1,3,5-tri­aza-7-phosphaadamantane (PTA), silver-PTA complex, cyanide ion

## Abstract

The title compound, [Ag(CN)(PTA)_3_]·4H_2_O, is a water-soluble discrete silver(I) complex where the silver center is coordinated by three phospho­rus atoms from distinct 1,3,5-tri­aza-7-phosphaadamantane (PTA, C_6_H_12_N_3_P) ligands and one carbon atom from a cyanide ion, forming a distorted tetra­hedral geometry. The asymmetric unit consisting of a [AgCN(PTA)_3/2_)] unit and two water mol­ecules. The Ag—P bond distances range from 2.4696 (4) to 2.4728 (6) Å, and the Ag—C bond distance is 2.168 (2) Å, while the bond angles around the silver center vary between 107.402 (13) and 111.07 (3)°, confirming its distorted tetra­hedral coordination environment. Inter­molecular O—H⋯N and O—H⋯O hydrogen bonds in the crystal packing of the title compound generate a two-dimensional supra­molecular architecture with a corrugated sheet-like topology that extends along the crystallographic *bc* plane.

## Abstract 2

The compound is a neutral discrete complex where the silver(I) center is coordinated by three phospho­rus atoms from three distinct PTA ligands and one carbon atom from the nitrile ion, resulting in a distorted tetra­hedral geometry around the Ag centre.

## Graphical abstract

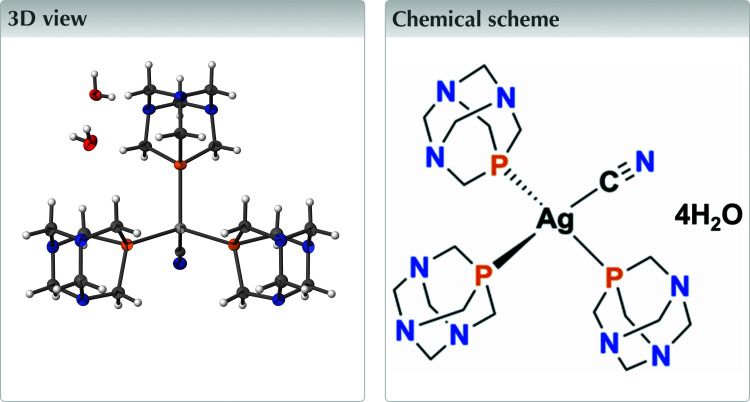

## Structure description

The cage-like, monodentate phosphine ligand 1,3,5-tri­aza-7-phosphaadamantane (PTA) and its derivatives have attracted significant attention due to their exceptional solubility in aqueous media, attributed to their unique structure featuring a soft phospho­rus donor and three hard nitro­gen atoms (Krogstad *et al.*, 2007 ▸). PTA typically coordinates metals *via* the phospho­rus atom in a monodentate fashion (PTA-κ*P*), while its nitro­gen atoms offer additional coordination possibilities that influence the structure and function of metal complexes (Darensbourg *et al.*, 1997 ▸). Silver(I) ions form stable and soluble complexes with PTA, enhancing their suitability for applications in catalysis (Phillips *et al.*, 2004 ▸), medicinal chemistry (Guerriero & Gonsalvi, 2021 ▸), and photoluminescence (Sierra-Martin *et al.*, 2018 ▸). Herein, the synthesis and crystal structure of the discrete monomeric complex [Ag(CN)(PTA)_3_]·4H_2_O, containing silver(I) coordinated by PTA and cyanide, are reported.

**Figure 1:**
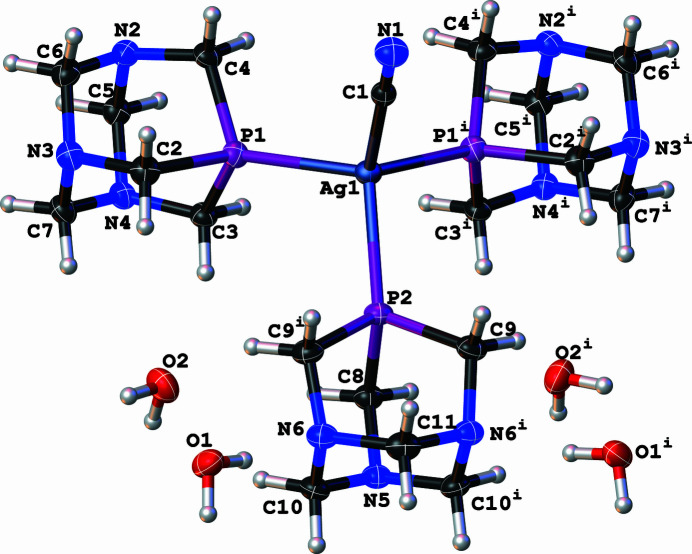
Mol­ecular structure of the title compound with ellipsoids drawn at the 50% probability level.

**Figure 2:**
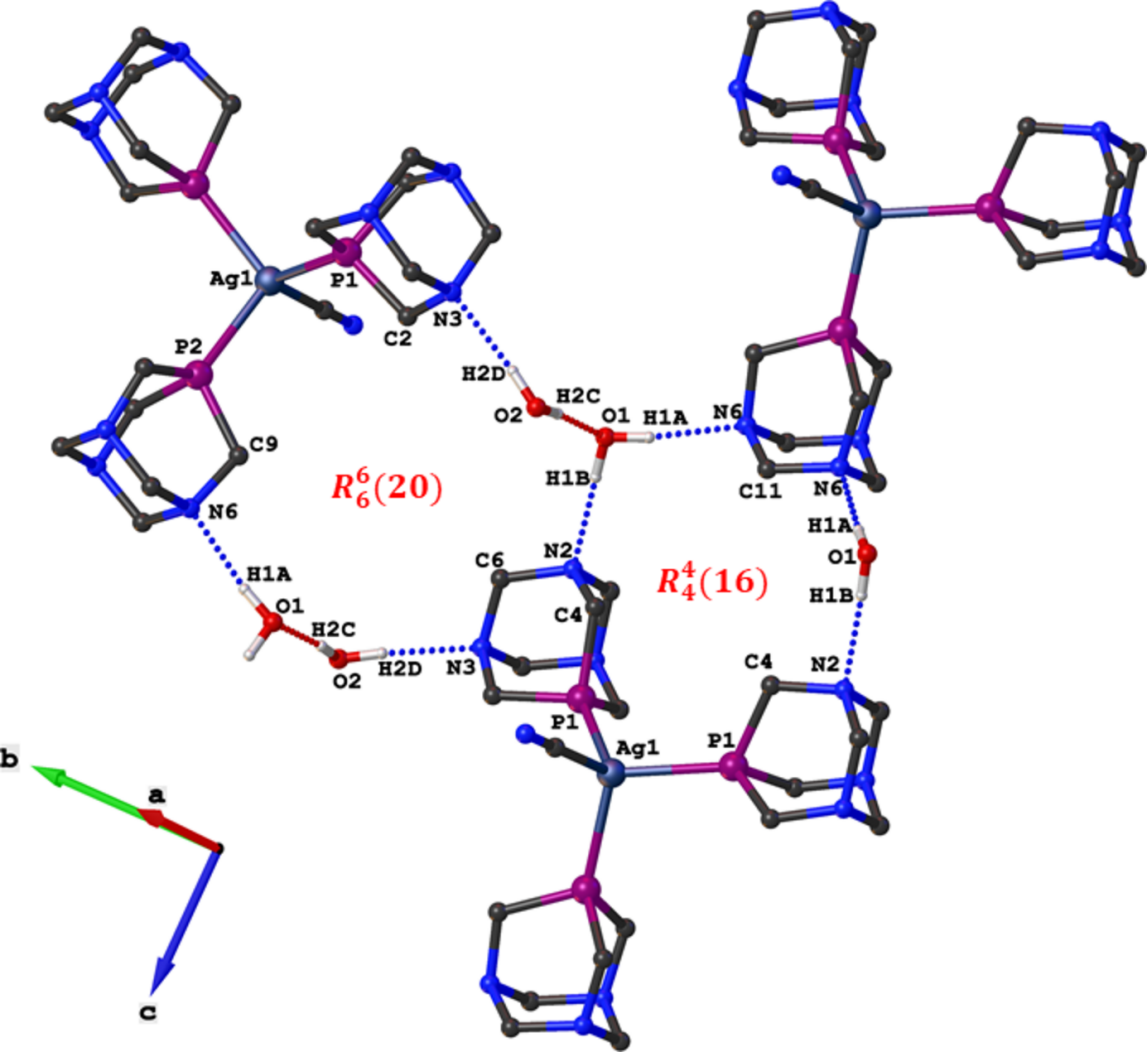
Representation of the inter­molecular O—H⋯O (red dotted lines) and O—H⋯N (blue dotted lines) hydrogen bond in the crystal packing of the title compound.

**Figure 3:**
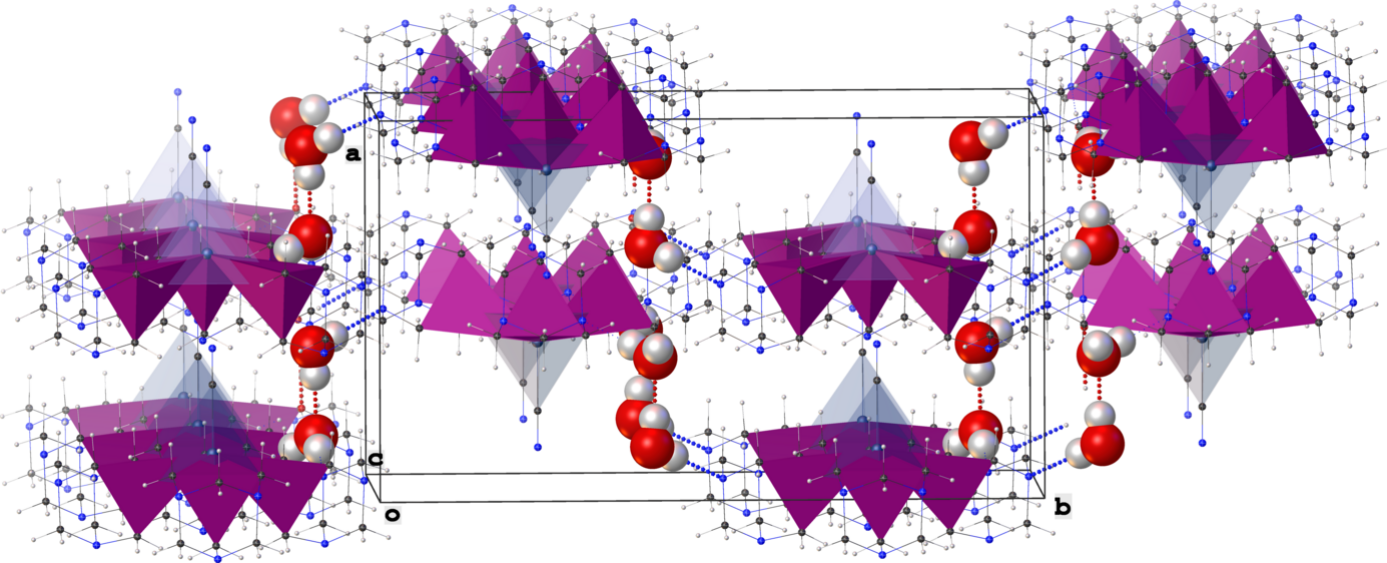
Representation of the 2-D supra­molecular structure of the title compound. The silver and phospho­rus atoms are drawn as blue- and purple-colored polyhedra, respectively, while the water mol­ecules are drawn using a space-filling model. The inter­molecular O—H⋯O and O—H⋯N hydrogen bonds are shown as red and blue dotted lines, respectively.

The asymmetric unit of title compound (Fig. 1 ▸) comprises a discrete [AgCN(PTA)_3/2_] unit and two water mol­ecules, with the full mol­ecule generated by the symmetry operation *x*, 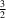 − *y*, *z*. It is a neutral discrete complex where the silver(I) center is coordinated by three phospho­rus atoms from three distinct PTA ligands and one carbon atom from the nitrile ion, resulting in a distorted tetra­hedral geometry around the Ag centre. The bond angles around Ag1 range from 107.402 (13) to 111.07 (3)°, with Ag—P bond distances between 2.4696 (4) and 2.4728 (6) Å and an Ag—C bond distance of 2.168 (2) Å. The crystal structure exhibits an intricate hydrogen-bonding network involving both water mol­ecules and the discrete [Ag(CN)(PTA)_3_] units. One water mol­ecule, O1, participates in two distinct hydrogen bonds, in one of which it links adjacent silver complexes through O—H⋯N hydrogen bonds involving nitro­gen atoms N2 and N6, forming hydrogen-bonded motifs described by the graph-set notation 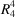
(16) (Table 1 ▸, Fig. 2 ▸). In the second, O1 acts as a hydrogen-bond acceptor while O2 functions as a donor, connecting through O—H⋯N hydrogen bonds to N3 of an adjacent [Ag(CN)(PTA)_3_] complex. This inter­action leads to the formation of a larger hydrogen-bonded ring which can be described by graph-set notation 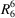
(20) (Fig. 2 ▸). Overall, these O—H⋯N and O—H⋯O hydrogen bonds generate a two-dimensional supra­molecular architecture with a corrugated sheet-like topology that extends along the crystallographic *bc* plane as shown in Fig. 3 ▸.

## Synthesis and crystallization

A methanol solution of PTA (3.84 g, 8.14 mmol) was mixed with aqueous KAg(CN)_2_ (3.00 g, 8.14 mmol). The solution volume was reduced by rotary evaporation, then aceto­nitrile (40 ml) was added to form a cloudy solution. The solvent was reduced using a rotary evaporator to give a white solid, which was isolated by vacuum filtration, washed with 2 × 5 ml of cold ethanol, and dried *in vacuo*. X-ray quality crystals were obtained by taking an aliquot of cloudy solution after the addition of aceto­nitrile and leaving it to stand overnight at room temperature.

## Refinement

Crystal data, data collection and structure refinement details are summarized in Table 2 ▸.

## Supplementary Material

Crystal structure: contains datablock(s) I. DOI: 10.1107/S2414314625009253/bx4039sup1.cif

Structure factors: contains datablock(s) I. DOI: 10.1107/S2414314625009253/bx4039Isup2.hkl

CCDC reference: 2496890

Additional supporting information:  crystallographic information; 3D view; checkCIF report

## Figures and Tables

**Table 1:** Hydrogen-bond geometry (Å, °)

*D*—H⋯*A*	*D*—H	H⋯*A*	*D*⋯*A*	*D*—H⋯*A*
O1—H1*A*⋯N6	0.87	1.97	2.8379 (19)	172
O1—H1*B*⋯N2^i^	0.87	1.98	2.8518 (18)	178
O2—H2*C*⋯O1^ii^	0.87	1.93	2.7931 (19)	174
O2—H2*D*⋯N3^iii^	0.87	2.01	2.8796 (19)	176

Symmetry codes: (i) 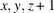; (ii) 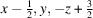; (iii) 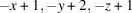.

**Table 2:** Experimental details

Crystal data	
Chemical formula	[Ag(CN)(C_6_H_12_N_3_P)_3_]·4H_2_O
*M*_r_	677.42
Crystal system, space group	Orthorhombic, *Pnma*
Temperature (K)	173
*a*, *b*, *c* (Å)	11.9813 (4), 20.8423 (7), 12.2359 (4)
*V* (Å^3^)	3055.52 (18)
*Z*	4
Radiation type	Mo *K*α
μ (mm^−1^)	0.86
Crystal size (mm)	0.35 × 0.26 × 0.2

Data collection	
Diffractometer	Bruker APEXII CCD
No. of measured, independent and observed [*I* > 2σ(*I*)] reflections	62216, 3898, 3438
*R*_int_	0.036
(sin θ/λ)_max_ (Å^−1^)	0.667

Refinement	
*R*[*F*^2^ > 2σ(*F*^2^)], *wR*(*F*^2^), *S*	0.024, 0.058, 1.11
No. of reflections	3898
No. of parameters	184
H-atom treatment	H-atom parameters constrained
Δρ_max_, Δρ_min_ (e Å^−3^)	0.43, −0.39

Computer programs: *COSMO* and *SAINT* (Bruker, 2009 ▸), *SHELXS* (Sheldrick, 2008 ▸), *SHELXL2018/3* (Sheldrick, 2015 ▸) and *OLEX2* (Dolomanov *et al.*, 2009 ▸).

## full crystallographic data

### Cyanidotris(1,3,5-triaza-7-phosphaadamantane)silver(I) tetrahydrate Crystal data

**Table:**

[Ag(CN)(C_6_H_12_N_3_P)_3_]·4H_2_O	*D*_x_ = 1.473 Mg m^−^^3^
*M**_r_* = 677.42	Mo *K*α radiation, λ = 0.71073 Å
Orthorhombic, *P**n**m**a*	Cell parameters from 9406 reflections
*a* = 11.9813 (4) Å	θ = 2.4–28.1°
*b* = 20.8423 (7) Å	µ = 0.86 mm^−^^1^
*c* = 12.2359 (4) Å	*T* = 173 K
*V* = 3055.52 (18) Å^3^	Plate, colourless
*Z* = 4	0.35 × 0.26 × 0.2 mm
*F*(000) = 1408	

### Cyanidotris(1,3,5-triaza-7-phosphaadamantane)silver(I) tetrahydrate Data collection

**Table:**

Bruker APEXII CCD diffractometer	*R*_int_ = 0.036
Graphite monochromator	θ_max_ = 28.3°, θ_min_ = 1.9°
φ and ω scans	*h* = −15→15
62216 measured reflections	*k* = −23→27
3898 independent reflections	*l* = −16→16
3438 reflections with *I* > 2σ(*I*)	

### Cyanidotris(1,3,5-triaza-7-phosphaadamantane)silver(I) tetrahydrate Refinement

**Table:**

Refinement on *F*^2^	0 restraints
Least-squares matrix: full	Hydrogen site location: mixed
*R*[*F*^2^ > 2σ(*F*^2^)] = 0.024	H-atom parameters constrained
*wR*(*F*^2^) = 0.058	*w* = 1/[σ^2^(*F*_o_^2^) + (0.0231*P*)^2^ + 1.851*P*] where *P* = (*F*_o_^2^ + 2*F*_c_^2^)/3
*S* = 1.11	(Δ/σ)_max_ = 0.001
3898 reflections	Δρ_max_ = 0.43 e Å^−^^3^
184 parameters	Δρ_min_ = −0.39 e Å^−^^3^

### Cyanidotris(1,3,5-triaza-7-phosphaadamantane)silver(I) tetrahydrate Special details

**Table:**

Geometry. All esds (except the esd in the dihedral angle between two l.s. planes) are estimated using the full covariance matrix. The cell esds are taken into account individually in the estimation of esds in distances, angles and torsion angles; correlations between esds in cell parameters are only used when they are defined by crystal symmetry. An approximate (isotropic) treatment of cell esds is used for estimating esds involving l.s. planes.
Refinement. Refinement of F^2^ against ALL reflections. The weighted R-factor wR and goodness of fit S are based on F^2^, conventional R-factors R are based on F, with F set to zero for negative F^2^. The threshold expression of F^2^ > 2sigma(F^2^) is used only for calculating R-factors(gt) etc. and is not relevant to the choice of reflections for refinement. R-factors based on F^2^ are statistically about twice as large as those based on F, and R- factors based on ALL data will be even larger.

### Cyanidotris(1,3,5-triaza-7-phosphaadamantane)silver(I) tetrahydrate Fractional atomic coordinates and isotropic or equivalent isotropic displacement parameters (Å^2^)

**Table:**

	*x*	*y*	*z*	*U*_iso_*/*U*_eq_	Occ. (<1)
Ag1	0.62465 (2)	0.750000	0.41337 (2)	0.02013 (6)	
P1	0.55217 (3)	0.84680 (2)	0.32110 (3)	0.02075 (9)	
P2	0.55002 (5)	0.750000	0.60179 (5)	0.02143 (13)	
N1	0.90084 (18)	0.750000	0.41775 (17)	0.0290 (5)	
N2	0.54207 (11)	0.92245 (7)	0.13404 (11)	0.0223 (3)	
N3	0.54339 (11)	0.98006 (7)	0.31065 (11)	0.0237 (3)	
N4	0.37430 (11)	0.92177 (6)	0.25146 (11)	0.0214 (3)	
N5	0.37219 (16)	0.750000	0.74784 (15)	0.0217 (4)	
N6	0.54111 (12)	0.80939 (7)	0.80538 (11)	0.0241 (3)	
C1	0.80555 (19)	0.750000	0.41643 (17)	0.0189 (4)	
C2	0.59015 (14)	0.92665 (8)	0.37527 (14)	0.0246 (4)	
H2A	0.563175	0.930191	0.451508	0.030*	
H2B	0.672493	0.930543	0.376499	0.030*	
C3	0.40053 (13)	0.86158 (8)	0.30910 (13)	0.0214 (3)	
H3A	0.365732	0.825328	0.269456	0.026*	
H3B	0.367502	0.863133	0.383219	0.026*	
C4	0.58916 (14)	0.86184 (8)	0.17668 (13)	0.0234 (3)	
H4A	0.671453	0.863213	0.169771	0.028*	
H4B	0.561692	0.825747	0.131485	0.028*	
C5	0.41875 (14)	0.92161 (8)	0.13962 (13)	0.0224 (3)	
H5A	0.389489	0.959593	0.100374	0.027*	
H5B	0.391112	0.882910	0.101303	0.027*	
C6	0.58279 (14)	0.97793 (8)	0.19676 (14)	0.0258 (4)	
H6A	0.558832	1.017712	0.159278	0.031*	
H6B	0.665409	0.977234	0.196821	0.031*	
C7	0.42017 (14)	0.97741 (8)	0.30966 (14)	0.0242 (3)	
H7A	0.393020	0.976580	0.386059	0.029*	
H7B	0.391326	1.017034	0.275111	0.029*	
C8	0.39835 (19)	0.750000	0.62980 (18)	0.0220 (5)	
H8A	0.364394	0.788376	0.595632	0.026*	0.5
H8B	0.364394	0.711624	0.595632	0.026*	0.5
C9	0.58800 (15)	0.68332 (9)	0.69428 (13)	0.0252 (4)	
H9A	0.560961	0.642493	0.662501	0.030*	
H9B	0.670341	0.680760	0.699484	0.030*	
C10	0.41789 (14)	0.80712 (8)	0.80178 (13)	0.0238 (3)	
H10A	0.389011	0.808843	0.877528	0.029*	
H10B	0.390437	0.845722	0.763055	0.029*	
C11	0.5815 (2)	0.750000	0.8573 (2)	0.0272 (5)	
H11A	0.557928	0.750000	0.934895	0.033*	
H11B	0.664079	0.750001	0.855846	0.033*	
O1	0.63144 (11)	0.91639 (6)	0.91827 (10)	0.0299 (3)	
H1A	0.599261	0.883175	0.888933	0.045*	
H1B	0.602530	0.918979	0.983341	0.045*	
O2	0.36431 (11)	0.91126 (6)	0.57555 (11)	0.0344 (3)	
H2C	0.292257	0.914947	0.581713	0.052*	
H2D	0.390923	0.943306	0.612940	0.052*	

### Cyanidotris(1,3,5-triaza-7-phosphaadamantane)silver(I) tetrahydrate Atomic displacement parameters (Å^2^)

**Table:**

	*U*^11^	*U*^22^	*U*^33^	*U*^12^	*U*^13^	*U*^23^
Ag1	0.01658 (9)	0.02286 (10)	0.02095 (9)	0.000	0.00045 (7)	0.000
P1	0.0166 (2)	0.0217 (2)	0.0239 (2)	0.00258 (17)	−0.00155 (16)	0.00100 (16)
P2	0.0180 (3)	0.0264 (3)	0.0200 (3)	0.000	0.0037 (2)	0.000
N1	0.0214 (11)	0.0348 (12)	0.0308 (11)	0.000	−0.0006 (9)	0.000
N2	0.0181 (7)	0.0238 (7)	0.0251 (7)	0.0004 (6)	0.0003 (6)	0.0019 (6)
N3	0.0189 (7)	0.0230 (7)	0.0290 (7)	−0.0010 (6)	−0.0025 (6)	−0.0016 (6)
N4	0.0157 (7)	0.0222 (7)	0.0263 (7)	0.0014 (6)	−0.0013 (5)	−0.0010 (5)
N5	0.0169 (10)	0.0270 (10)	0.0213 (9)	0.000	0.0019 (7)	0.000
N6	0.0198 (7)	0.0299 (8)	0.0226 (6)	−0.0020 (6)	0.0016 (5)	−0.0026 (6)
C1	0.0202 (12)	0.0193 (11)	0.0171 (9)	0.000	−0.0007 (8)	0.000
C2	0.0194 (8)	0.0275 (9)	0.0270 (8)	−0.0016 (7)	−0.0054 (7)	−0.0006 (7)
C3	0.0160 (8)	0.0236 (8)	0.0246 (8)	−0.0006 (6)	0.0012 (6)	−0.0008 (6)
C4	0.0168 (8)	0.0266 (9)	0.0268 (8)	0.0035 (7)	0.0019 (6)	0.0000 (7)
C5	0.0183 (8)	0.0249 (8)	0.0239 (8)	0.0010 (7)	−0.0040 (6)	0.0011 (6)
C6	0.0202 (8)	0.0256 (9)	0.0315 (9)	−0.0034 (7)	0.0003 (7)	0.0024 (7)
C7	0.0201 (8)	0.0222 (8)	0.0301 (8)	0.0026 (7)	−0.0010 (7)	−0.0034 (7)
C8	0.0177 (11)	0.0266 (12)	0.0215 (10)	0.000	−0.0004 (9)	0.000
C9	0.0206 (8)	0.0303 (9)	0.0247 (8)	0.0050 (7)	0.0037 (7)	0.0010 (7)
C10	0.0199 (8)	0.0279 (9)	0.0235 (8)	0.0022 (7)	0.0031 (7)	−0.0027 (7)
C11	0.0204 (12)	0.0380 (14)	0.0231 (11)	0.000	−0.0034 (10)	0.000
O1	0.0335 (7)	0.0293 (7)	0.0268 (6)	−0.0078 (6)	0.0049 (5)	0.0000 (5)
O2	0.0333 (7)	0.0291 (7)	0.0407 (8)	0.0006 (6)	0.0008 (6)	−0.0107 (6)

### Cyanidotris(1,3,5-triaza-7-phosphaadamantane)silver(I) tetrahydrate Geometric parameters (Å, º)

**Table:**

Ag1—P1^i^	2.4696 (4)	N6—C11	1.4732 (19)
Ag1—P1	2.4696 (4)	C2—H2A	0.9900
Ag1—P2	2.4728 (6)	C2—H2B	0.9900
Ag1—C1	2.168 (2)	C3—H3A	0.9900
P1—C2	1.8484 (17)	C3—H3B	0.9900
P1—C3	1.8486 (16)	C4—H4A	0.9900
P1—C4	1.8486 (17)	C4—H4B	0.9900
P2—C8	1.849 (2)	C5—H5A	0.9900
P2—C9^i^	1.8492 (17)	C5—H5B	0.9900
P2—C9	1.8492 (17)	C6—H6A	0.9900
N1—C1	1.142 (3)	C6—H6B	0.9900
N2—C4	1.479 (2)	C7—H7A	0.9900
N2—C5	1.479 (2)	C7—H7B	0.9900
N2—C6	1.471 (2)	C8—H8A	0.9900
N3—C2	1.476 (2)	C8—H8B	0.9900
N3—C6	1.472 (2)	C9—H9A	0.9900
N3—C7	1.477 (2)	C9—H9B	0.9900
N4—C3	1.473 (2)	C10—H10A	0.9900
N4—C5	1.468 (2)	C10—H10B	0.9900
N4—C7	1.468 (2)	C11—H11A	0.9900
N5—C8	1.478 (3)	C11—H11B	0.9900
N5—C10	1.467 (2)	O1—H1A	0.8700
N5—C10^i^	1.467 (2)	O1—H1B	0.8700
N6—C9^i^	1.479 (2)	O2—H2C	0.8700
N6—C10	1.478 (2)	O2—H2D	0.8700
			
P1^i^—Ag1—P1	109.55 (2)	N2—C4—P1	113.00 (11)
P1^i^—Ag1—P2	107.402 (13)	N2—C4—H4A	109.0
P1—Ag1—P2	107.402 (13)	N2—C4—H4B	109.0
C1—Ag1—P1	111.07 (3)	H4A—C4—H4B	107.8
C1—Ag1—P1^i^	111.07 (3)	N2—C5—H5A	108.8
C1—Ag1—P2	110.21 (6)	N2—C5—H5B	108.8
C2—P1—Ag1	119.02 (6)	N4—C5—N2	113.91 (13)
C2—P1—C3	96.91 (8)	N4—C5—H5A	108.8
C2—P1—C4	97.53 (8)	N4—C5—H5B	108.8
C3—P1—Ag1	121.21 (5)	H5A—C5—H5B	107.7
C3—P1—C4	97.55 (7)	N2—C6—N3	114.28 (14)
C4—P1—Ag1	119.42 (6)	N2—C6—H6A	108.7
C8—P2—Ag1	121.88 (8)	N2—C6—H6B	108.7
C8—P2—C9	97.38 (7)	N3—C6—H6A	108.7
C9—P2—Ag1	118.79 (5)	N3—C6—H6B	108.7
C9^i^—P2—Ag1	118.79 (5)	H6A—C6—H6B	107.6
C9^i^—P2—C8	97.38 (7)	N3—C7—H7A	108.7
C9^i^—P2—C9	97.46 (11)	N3—C7—H7B	108.7
C4—N2—C5	110.78 (13)	N4—C7—N3	114.06 (13)
C6—N2—C4	111.15 (13)	N4—C7—H7A	108.7
C6—N2—C5	108.45 (13)	N4—C7—H7B	108.7
C2—N3—C7	110.83 (13)	H7A—C7—H7B	107.6
C6—N3—C2	111.27 (14)	P2—C8—H8A	109.0
C6—N3—C7	108.15 (13)	P2—C8—H8B	109.0
C5—N4—C3	111.53 (12)	N5—C8—P2	112.93 (15)
C7—N4—C3	111.14 (13)	N5—C8—H8A	109.0
C7—N4—C5	108.55 (13)	N5—C8—H8B	109.0
C10^i^—N5—C8	111.13 (11)	H8A—C8—H8B	107.8
C10—N5—C8	111.13 (12)	P2—C9—H9A	109.0
C10—N5—C10^i^	108.47 (18)	P2—C9—H9B	109.0
C10—N6—C9^i^	110.82 (13)	N6^i^—C9—P2	113.07 (12)
C11—N6—C9^i^	111.00 (15)	N6^i^—C9—H9A	109.0
C11—N6—C10	108.29 (15)	N6^i^—C9—H9B	109.0
N1—C1—Ag1	179.82 (19)	H9A—C9—H9B	107.8
P1—C2—H2A	108.9	N5—C10—N6	114.35 (14)
P1—C2—H2B	108.9	N5—C10—H10A	108.7
N3—C2—P1	113.18 (11)	N5—C10—H10B	108.7
N3—C2—H2A	108.9	N6—C10—H10A	108.7
N3—C2—H2B	108.9	N6—C10—H10B	108.7
H2A—C2—H2B	107.8	H10A—C10—H10B	107.6
P1—C3—H3A	109.0	N6—C11—N6^i^	114.33 (19)
P1—C3—H3B	109.0	N6^i^—C11—H11A	108.7
N4—C3—P1	112.93 (11)	N6—C11—H11A	108.7
N4—C3—H3A	109.0	N6—C11—H11B	108.7
N4—C3—H3B	109.0	N6^i^—C11—H11B	108.7
H3A—C3—H3B	107.8	H11A—C11—H11B	107.6
P1—C4—H4A	109.0	H1A—O1—H1B	104.5
P1—C4—H4B	109.0	H2C—O2—H2D	104.5
			
Ag1—P1—C2—N3	178.57 (9)	C6—N2—C4—P1	60.19 (15)
Ag1—P1—C3—N4	179.99 (8)	C6—N2—C5—N4	−55.12 (17)
Ag1—P1—C4—N2	−178.41 (9)	C6—N3—C2—P1	−59.84 (16)
Ag1—P2—C8—N5	180.000 (1)	C6—N3—C7—N4	55.62 (18)
Ag1—P2—C9—N6^i^	177.87 (9)	C7—N3—C2—P1	60.54 (16)
C2—P1—C3—N4	49.85 (12)	C7—N3—C6—N2	−55.30 (18)
C2—P1—C4—N2	−48.90 (13)	C7—N4—C3—P1	−61.08 (15)
C2—N3—C6—N2	66.65 (17)	C7—N4—C5—N2	55.58 (17)
C2—N3—C7—N4	−66.60 (18)	C8—P2—C9—N6^i^	−49.37 (14)
C3—P1—C2—N3	−49.83 (13)	C8—N5—C10—N6	−67.05 (19)
C3—P1—C4—N2	49.15 (13)	C9^i^—P2—C8—N5	−49.27 (6)
C3—N4—C5—N2	−67.19 (17)	C9—P2—C8—N5	49.28 (6)
C3—N4—C7—N3	67.02 (17)	C9^i^—P2—C9—N6^i^	49.11 (16)
C4—P1—C2—N3	48.78 (13)	C9^i^—N6—C10—N5	66.84 (19)
C4—P1—C3—N4	−48.73 (12)	C9^i^—N6—C11—N6^i^	−66.9 (2)
C4—N2—C5—N4	67.12 (17)	C10—N5—C8—P2	60.44 (12)
C4—N2—C6—N3	−66.84 (17)	C10^i^—N5—C8—P2	−60.44 (12)
C5—N2—C4—P1	−60.46 (15)	C10^i^—N5—C10—N6	55.4 (2)
C5—N2—C6—N3	55.17 (18)	C10—N6—C11—N6^i^	54.9 (2)
C5—N4—C3—P1	60.20 (15)	C11—N6—C10—N5	−55.13 (18)
C5—N4—C7—N3	−55.98 (17)		

Symmetry code: (i) *x*, −*y*+3/2, *z*.

### Cyanidotris(1,3,5-triaza-7-phosphaadamantane)silver(I) tetrahydrate Hydrogen-bond geometry (Å, º)

**Table:**

*D*—H···*A*	*D*—H	H···*A*	*D*···*A*	*D*—H···*A*
O1—H1*A*···N6	0.87	1.97	2.8379 (19)	172
O1—H1*B*···N2^ii^	0.87	1.98	2.8518 (18)	178
O2—H2*C*···O1^iii^	0.87	1.93	2.7931 (19)	174
O2—H2*D*···N3^iv^	0.87	2.01	2.8796 (19)	176

Symmetry codes: (ii) *x*, *y*, *z*+1; (iii) *x*−1/2, *y*, −*z*+3/2; (iv) −*x*+1, −*y*+2, −*z*+1.
